# Metastatic Melanoma of the Gallbladder in an Asymptomatic Patient

**DOI:** 10.1155/2017/1767418

**Published:** 2017-08-22

**Authors:** Asad Khan, Sejal Patel, Daniel J. Zaccarini, Mary McGrath

**Affiliations:** ^1^SUNY Upstate Medical University, Syracuse, NY 13210, USA; ^2^Department of Radiology, SUNY Upstate Medical University, Syracuse, NY 13210, USA; ^3^Department of Pathology, SUNY Upstate Medical University, Syracuse, NY 13210, USA; ^4^Department of Nuclear Medicine, SUNY Upstate Medical University, Syracuse, NY 13210, USA

## Abstract

Malignant Melanoma (MM) is among the most dangerous malignancies with some of the least known survival rates. Melanoma most commonly metastasizes to regional lymph nodes, the lungs, and brain. Metastatic disease of the gallbladder (GB) is exceptionally rare making it difficult to diagnose. The fact that typically patients do not present until they are symptomatic—only after widespread metastatic disease has already occurred—is further complicating the diagnosis of MM of the GB. For this reason, MM of the GB is rarely discovered in living patients. In fact, review of the literature showed only 40 instances in which metastatic disease of the GB was reported in living patients. We describe the presentation and management of a patient who had metastatic disease of the GB. However, our case is unique because his malignancy was discovered incidentally while he was asymptomatic. He was successfully treated with an open cholecystectomy. We have presented this case in order to make the necessity of meticulous investigation of potential metastasis in patients with a known history of cutaneous melanoma clear.

## 1. Introduction

Metastatic melanoma is among the most aggressive malignancies with some of the least known survival rates with a median survival of 6–10 months after surgery and 18% survival at five years [1]. Melanoma most commonly metastasizes to regional lymph nodes, the lungs, and brain [2, 3]. Metastatic disease of the gastrointestinal (GI) system is unusual and only occurs in 2–4% of patients [4]. Within the GI system metastatic disease most commonly occurs in the intestines, colon, and the stomach [4].

Metastatic disease of the gallbladder is rare and associated with a poor prognosis with a mean survival rate of 8.4 months [5]. Furthermore, patients typically do not report symptoms during their lifetime because involvement of the gallbladder seldom produces symptoms [6]. For this reason, melanoma of the gallbladder is rarely discovered in living patients [6]. A review of the literature shows only 40 instances in which metastatic disease of the gallbladder was reported in living patients [7].

We herein report a case of a 57-year-old gentleman with a history of cutaneous melanoma of the shoulder that was incidentally discovered to have metastatic melanoma of the gallbladder, which was successfully treated with open cholecystectomy. Our patient's case is particularly unique due to discovery of the mass while being alive and asymptomatic.

## 2. Case Presentation

A 57-year-old gentleman was diagnosed with depth of 2.71 mm, Clark level IV, ulcerated cutaneous melanoma of the left shoulder and back, which showed perivascular invasion and superficial spreading. Preoperative lymphoscintigraphy showed a focus of increased activity in the left axilla, which was removed and negative on final pathology for metastatic melanoma.

The patient was treated with wide local excision and a total of four sentinel lymph nodes were removed, which were negative for malignancy. He also underwent metastatic workup including CT of the chest, abdomen, and pelvis that was negative for any distant metastasis; however, it did show some hepatic cysts that warranted follow-up imaging.

**Figure 1:**
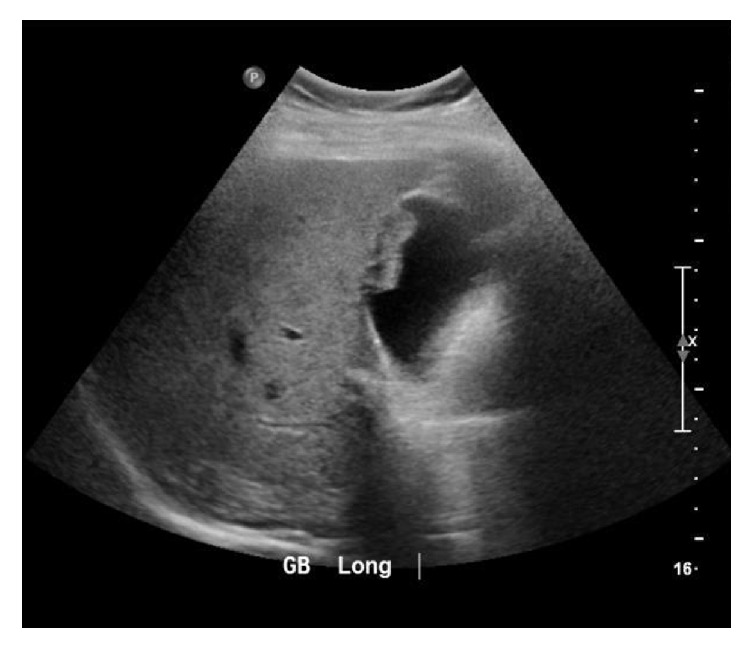
Ultrasound of the gallbladder demonstrating an irregular lesion along the gallbladder wall measuring 2.9 × 1.0 cm.

**Figure 2:**
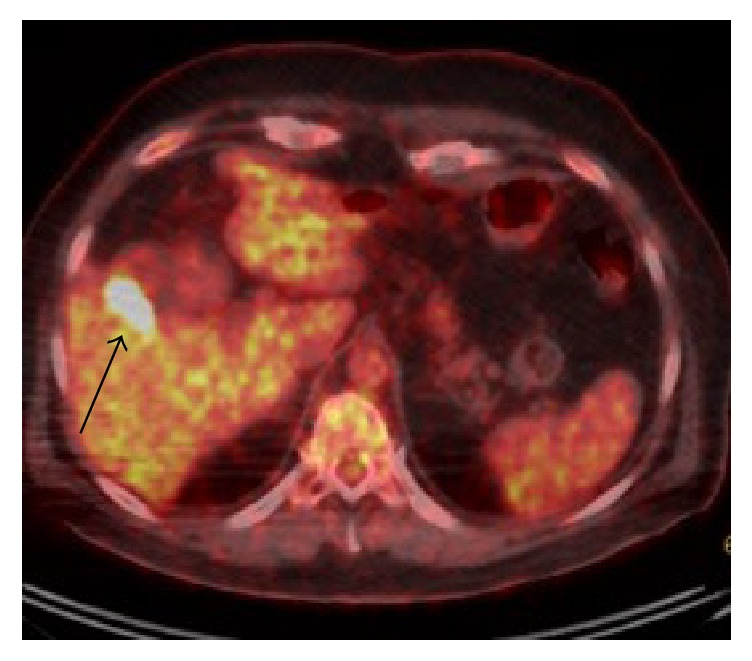
Fused PET/CT axial image demonstrating intense increased activity in the gallbladder (black arrow).

**Figure 3:**
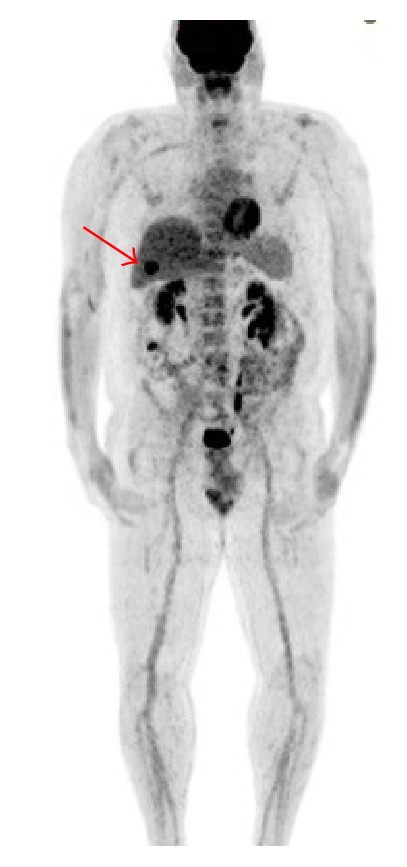
Whole body MIP image showing abnormal focal activity in the region of the gallbladder (red arrow).

**Figure 4:**
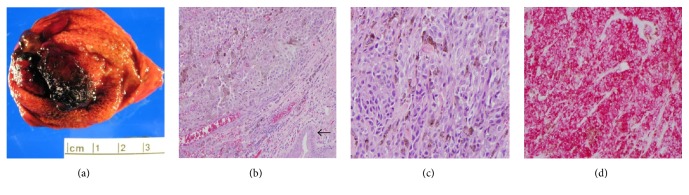
(a) Gallbladder mucosa with dark brown pigmented polypoid lesion measuring 2.7 × 1.5 × 1.3 cm. (b) Sheets of malignant epithelioid cells are seen with eosinophilic cytoplasm and prominent nucleoli. Gallbladder epithelium can be seen (arrow). (c) Higher power showing malignant cells with hyperchromatic large atypical nuclei with eosinophilic cytoplasm and scattered brown pigment. (d) Immunohistochemistry for melanocytic marker HMB-45 showing diffuse and strong staining.

A sonographic exam of the right upper quadrant of the abdomen was performed for evaluation of liver cysts and incidentally noted an irregular lesion along the gallbladder wall (Figure 1). A follow-up MRI was recommended, which showed multiple hepatic cysts and a nonspecific gallbladder mass measuring 2.8 cm in transverse diameter. The decision was then made to proceed with PET scan and MRI of the brain for staging given the history of high-risk melanoma. The PET scan showed a focus of increased metabolic activity with maximum SUV of 22.1 within the gallbladder in the region of abnormality visualized on the ultrasound and MRI (Figures 2 and 3). The patient underwent cholecystectomy with final pathology remarkable for metastatic melanoma (Figure 4).

## 3. Discussion

Melanoma is a cancer of melanocytes, which are dendritic cells responsible for producing melanin, and represents approximately <5% of all skin cancers [8]. It is associated with high mortality due to the potential for wide spread metastatic disease. Once metastatic disease has occurred, prognosis is very poor with a mean survival rate of approximately 8.4 months [5].

Autopsy reports show the prevalence of metastatic disease of the gallbladder is between 15 and 20% [6]. Melanoma is the most common metastatic malignancy of the gallbladder, accounting for about 50–67% [9]. Despite these statistic data, most patients are asymptomatic and do not present until widespread disease has occurred [10–12]. Thus accurate diagnosis requires a strong degree of suspicion. Among the symptomatic patients, acute cholecystitis is the most common presentation, followed by jaundice and obstruction of the common bile duct [13]. Incidences of hemobilia and biliary fistula have also been reported [14].

Ultrasound is the preferred imaging modality for evaluation of the gallbladder in symptomatic patients or in patients in whom metastatic disease of the gallbladder is suspected [15]. Typical ultrasound findings include a sole or multiple infiltrative polypoid lesions not showing acoustic shadow, larger than 1 cm, and attached to the GB wall [4]. Doppler ultrasound has been shown to be useful in detecting the presence of remarkably increased blood flow within the lesions [16].

While the examination of choice in detecting metastatic melanoma of the gallbladder is straightforward, the treatment is much less definitive. There is limited experience in the management of metastatic melanoma of the gallbladder due to its rarity. In general, patients with disease limited to the GB should be aggressively treated with surgery with a goal of avoiding symptoms or tumor complications and significant improvement in survival and prognosis [1]. One study done by Dong et al. found that patients who had surgery for isolated gallbladder metastases had a 100% survival at 1 year compared with 0% for those with unresectable tumors with multiple metastases [10]. When considering surgical approach, open cholecystectomy is preferred over laparoscopic technique, which has been associated with port site recurrence [1, 17].

One promising avenue for the future in the treatment of metastatic melanoma is chemoimmunotherapy. One study documented the use of high-dose interleukin-2 in tumor remission in approximately 15% of patients [18]. However, the use of interleukin-2 is limited on a large scale due to toxicity and difficulties with its administration. The BRAF inhibitor, vemurafenib, has demonstrated improved progression-free and overall survival compared with chemotherapy in a randomized trial [19]. The use of vemurafenib in patients who are BRAF V600E positive has become the standard of care [19]. Other studies have shown that combining the use of BRAF inhibitors with the use of MEK inhibitors lowers the incidence of adverse reactions, prolongs disease-free survival, and delays the resistance that has been seen in BRAF inhibitor use alone [20].

## 4. Conclusion

Metastatic melanoma is among the most ominous malignancies due to its aggressive course and poor prognosis. We have presented this case in order to make the necessity of meticulous investigation of potential metastases in patients with a known history of cutaneous melanoma clear. Metastatic melanoma of the gallbladder is considered an especially rare event and diagnosis is difficult because most cases of gallbladder metastasis are asymptomatic. Various studies indicate that the key to prolonging survival is aggressive treatment once metastatic disease is discovered. Acceptable treatment options include cholecystectomy and chemoimmunotherapy.
